# The 2013 measles outbreak in Sri Lanka: experience from a rural district and implications for measles elimination goals

**DOI:** 10.1186/s40249-015-0084-7

**Published:** 2015-11-30

**Authors:** Niroshana Jathun Dahanayaka, Sithumini Pahalagamage, Ranjan Madushanka Ganegama, Prasanna Weerawansa, Suneth Buddhika Agampodi

**Affiliations:** Department of Medicine, Faculty of Medicine and Allied Sciences, Rajarata University of Sri Lanka, Saliyapura, Sri Lanka; Tropical Disease Research Unit, Faculty of Medicine and Allied Sciences, Rajarata University of Sri Lanka, Saliyapura, Sri Lanka; University Medical Unit, Teaching Hospital, Anuradhapura, Sri Lanka; Department of Community Medicine, Faculty of Medicine and Allied Sciences, Rajarata University of Sri Lanka, Saliyapura, Sri Lanka

**Keywords:** Measles, Measles elimination, Rural districts, Anuradhapura, Sri Lanka

## Abstract

**Background:**

Sri Lanka was the first country in the Southeast Asian region to achieve its measles elimination goal in 2011. In 2012, the measles immunization schedule changed from a measles vaccine at 9 months to a measles, mumps and rubella vaccine at 12 months. However in 2013, Sri Lanka reported its worst recent outbreak of measles. This study investigated a part of this outbreak in order to describe its epidemiology.

**Methods:**

A prospective study was carried out at the university medical unit of the Teaching Hospital, Anuradhapura (THA), the third largest hospital in Sri Lanka, from October 2013 until March 2014. An epidemiological profile of patients was constructed, case confirmation was done on all suspected cases and the basic demographic details of these suspected cases were obtained from the available records.

**Results:**

From January 2013 to March 2014, 101 measles suspects were admitted to the THA. Until June 2013, all suspected cases were aged below 12 months of age. During the study period (15 months), the total number of patients aged below 9 months, 9 to 12 months, 1 to 11 years, 12–29 years and over 29 years were 10 (9.9 %), 11 (10.9 %), 6 (5.9 %), 37 (36.6 %) and 36 (35.6 %), respectively (data missing-1). Out of the 33 patients clinically suspected, 32 tested positive for measles. Common clinical features included: fever (*n* = 33, 100 %), maculopapular rash (*n* = 33), conjunctivitis (*n* = 31), posterior cervical lymphadenopathy (*n* = 23) and Koplik’s spots (*n* = 8). Features suggestive of pneumonia were observed among 30 (90.9 %) patients and 26 (78.8 %) had diarrhoea. Two patients (6.1 %) who developed severe pneumonia received care at an intensive care unit due to respiratory difficulties. Out of 33 patients, 15 (45.5 %) had prior immunization for measles, two (6.1 %) reported that they never had a measles immunization and 16 (48.5 %) were unsure about their immunization status. Out of those who reported they were previously immunized, 11 (73.3 %) belonged to the age group of 12–29 years.

**Conclusion:**

Because the first cases of this outbreak were infants, an increase in susceptible infants due to the change in the vaccine schedule could partly explain the outbreak.

**Electronic supplementary material:**

The online version of this article (doi:10.1186/s40249-015-0084-7) contains supplementary material, which is available to authorized users.

## Multilingual abstracts

Please see Additional file 1 for translations of the abstract into the six official working languages of the United Nations.

## Background

Measles is one of the most contagious human diseases. Before the introduction of measles-containing vaccines (MCVs), 90 % of children had measles during the first 15 years of their lives. In 1980, the global number of deaths attributed to measles was exceeding 2.5 million [1]. The first MCV was licensed in the USA in 1963 based on the pioneering work of Enders [2, 3]. This vaccine has since become one of the most effective interventions that mankind has ever invented [4].

After the successful eradication of small pox, measles elimination was the number one priority in vaccine preventable disease control programmes worldwide. A steady increase of MCV coverage and the subsequent decline of measles incidence has been observed globally over the past two decades [5]. In 2008, however, there was a worldwide resurgence of measles, mainly affecting 28 Sub-Saharan African countries [6–10]. More than 200,000 cases and more than 1400 deaths were reported during this outbreak [11].

Sri Lanka’s measles control programme has achieved many of its goals since setting its measles elimination targets in 1984. The annual incidence of measles in the country between 1971 and 1980 varied from 12 to 49 per 100,000 population. In August 1984, the measles vaccine was introduced into the expanded programme on immunization (EPI). After this, several local outbreaks of measles [12] were observed, but the annual incidence of measles declined, up until 1999. A measles epidemic then hit, lasting from October 1999 to June 2000; more than 15,000 cases and five deaths were reported. Nearly 54 % of the cases were ≥15 years of age at the time of disease onset. The highest morbidity was observed in the age group of <9 months (114 cases per 100,000 population), followed by the 15–19 age group (87 per 100,000 population) [13]. After the 1999–2000 measles outbreak, a two-dose schedule of the measles vaccination was introduced into the EPI. There has been a decline in annual measles incidence since then, reaching the elimination target of <5 per million population in 2011.

In the second quarter of 2013, however, Sri Lanka experienced another island-wide measles outbreak. It started in Colombo, soon spreading all over the country. Even though this outbreak was the second largest the country has seen in 20 years, scientific research to describe this outbreak is scarce. The purpose of this study is to describe the epidemiology of the 2013 measles outbreak in Sri Lanka.

## Methods

### Study site and design

To describe the spatio-temporal distribution of measles, we used secondary data on measles patients admitted to the Teaching Hospital, Anuradhapura (THA). The THA is the third' largest hospital in Sri Lanka and the main referral hospital in the Anuradhapura district. It has three medical and three paediatric units. At the THA, infectious control nurses are responsible for the surveillance of communicable diseases. They collect basic information from all inpatients with suspected communicable diseases.

A descriptive cross-sectional study was carried out on suspected measles patients admitted to the university medical unit (UMU) at the THA from October 2013, in order to describe their clinical and bio-chemical profiles and complications. Inclusion criteria were based on the clinician’s diagnosis. If the treating physician tentatively diagnosed measles in a patient, he/she was included in the study.

### Data collection

Following the tentative diagnosis, 3 ml of venous blood was obtained and sent to the Medical Research Institute (MRI) of Sri Lanka for disease confirmation, using the measles IgM enzyme-linked immunosorbent assay (ELISA) test. On admission, suspected measles cases underwent clinical examination by two investigators to document the patients’ positive and negative clinical features. The patients were then followed up until they were discharged from hospital. Because measles is a vaccine-preventable disease, special case report forms are available to be used by health professionals for disease investigation. We used these interviewer-administered forms to collect patients’ basic socio-demographic data. All clinical data were carefully documented using a clinical data checklist under the supervision of the treating physician. Details on blood biochemistry of the patients were extracted from the patient’s records (bed-head tickets).

### Data analysis

Only descriptive analysis was performed using percentages and proportions.

### Ethical clearance

Informed consent was obtained from all eligible patients prior to commencement of the study. Ethical clearance for the study was obtained from the Ethics Review Committee of the Faculty of Medicine and Allied Sciences, Rajarata University of Sri Lanka.

## Results

**Fig. 1:**
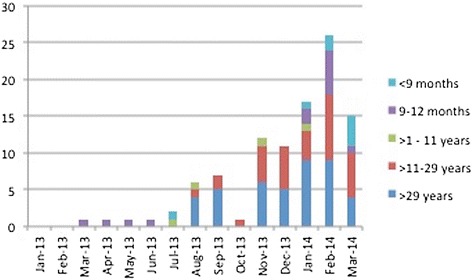
Temporal distribution of suspected measles cases admitted to the THA from January 2013 to March 2014, by age group

One hundred and one patients with suspected measles were admitted to the THA within the 15-month period between January 2013 and March 2014. The total numbers of patients aged below 9 months, 9 to 12 months, 1 to 11 years, 12–29 years and over 29 years were 10 (9.9 %), 11 (10.9 %), 6 (5.9 %), 37 (36.6 %) and 36 (35.6 %), respectively (age was missing in one patient admitted to paediatric ward). Of the total participants, 52 (51.5 %) were males. There were no suspected measles patients admitted during the first two months of 2013 (see Fig. 1). There was one admission each in the following 4 months, all of whom were in the 9 to 12 months age group.

A clinical study with case confirmation was carried out in the UMU. A total of 33 patients with suspected measles were admitted to the unit, from the last week of October 2013 to the end of March 2014. Serum samples from 32 patients were sent to the MRI for disease confirmation and all 32 (100 %) tested positive for IgM measles antibodies.

The mean age was 27.64 ± 7.38 and the youngest patient was 16 (admission to the UMU were patients older than 13 years). Out of the 33 patients, 21 (63.6 %) were males.

All patients had fever on admission (see Table 1). Maculopapular rash was observed in 27 patients on admission. We specifically looked for typical Koplik’s spots; it was observed in eight patients. Two patients (6.1 %) who developed severe pneumonia received care at an intensive care unit (ICU) due to respiratory difficulties. Both of them had bi-lateral patchy opacifications in the chest radiograph and received non-invasive positive pressure ventilation together with broad-spectrum antibiotics. The first patient had a dramatic recovery within 24 h, but the second patient was in the ICU for 72 h.

**Table 1:** Clinical profile of 33 measles patients admitted to the UMU THA, 2013–14

Clinical features	N(%)
Fever on admission	33(100)
Rash on admission	27(81.8)
Rash developed after admission	6(18.2)
Generalized rash	31(93.9)
First appearance of rash	
Face	17(51.5)
Trunk	14(42.4)
Extremities (arms and legs)	2(6.1)
Cough	33(100)
Conjunctivitis	31(93.9)
Coryza	29(87.9)
Diarrhoea	26(78.8)
Posterior cervical lymphadenopathy	23(69.7)
Features of pneumonia	30(90.9)
Hepatomegaly	14(42.4)
Koplik’s spots	8(24.2)

All but two patients had normal white cell count (WCC) (see Table 2). A differential count revealed neutrophils predominance in all patients. The mean platelet count was 159 ± 50.2 × 10^3^/μL and 17 patients had a platelet count of less than 150 × 10^3^/μL.

**Table 2:** Blood and urine biochemistry of the 33 measles patients admitted to the UMU THA, 2013–14

	Mean ± SD
Haemoglobin (mg/dL)	13.4 ± 1.3
WCC (10^3^/mm^3^)	6.1 ± 2.1
WCC range (10^3^/μL)	2.3–13.3
Neutrophil (%)	80.7 ± 6.9
Neutrophil range (%)	61–94 %
Lymphocytes (%)	12.8 ± 6.2
Platelet count (10^3^/ μL)	159 ± 50.2
Platelet count (<150 × 10^3^/ μL)	17(51.5 %)
SGOT (IU/L)	138.1 ± 81.2
SGOT range (IU/L)	48–326
SGPT (IU/L)	137.6 ± 91.5
SGPT range (IU/L)	42–316
ESR (mm in first hour)	18.8 ± 9.1
CRP	26.8 ± 20.2
Proteinuria	12(36.3)

*WCC* white cell count, *SGOT* serum glutamic oxaloacetic transaminase, *SGPT* serum glutamic-pyruvic transaminase, *ESR* erythrocyte sedimentation rate, *CRP* C-reactive protein

A late presentation to hospital among patients was observed and the median duration of fever on admission was 5 days (IQR: 4 to 7 days). The total duration of illness (onset of the first symptom until discharge) ranged from 5 to 16 days, with a median of 8 days. The median duration of hospital stay was 4 days (range: 2 to 7 days).

Out of the 33 patients admitted to the UMU THA, 15 (45.5 %) had prior immunization for measles, two (6.1 %) reported that they never had a measles immunization and 16 (48.5 %) were unsure about their immunization status. Out of those who reported that they were previously immunized, 11 (73.3 %) belonged to age group of 12–29 years. Five patients gave a contact history of measles over the last 4 weeks and two reported that a family member had fever and rash. Only one patient said he/she lived in a semi-permanent structure with poor hygiene and sanitary conditions.

## Discussion

In this study, we investigated a part of Sri Lanka’s most recent measles outbreak. The study showed that the first wave of patients during this outbreak in Anuradhapura comprised infants aged below 12 months. Furthermore, our detailed investigation of adult patients revealed that the majority showed signs of developing severe complications such as pneumonia, with a few requiring intensive care.

As part of the measles elimination programme, a MCV was introduced into Sri Lanka’s EPI in 1984 as a single-dose vaccine administered to infants aged 9 months. Therefore, individuals aged 29 years or above have not had a routine vaccination for measles. Most of the individuals in the 11–29 age group only had a single dose of the MCV, as the guideline for a second dose was only added to the EPI schedule (measles-rubella vaccine at 3 years) in 2002, following the island-wide measles outbreak of 1999–2000. In 2012, the EPI schedule was revised again, with the introduction of the measles, mumps and rubella vaccine to be administered to babies aged 12 months.

The 2013 outbreak of measles in Anuradhapura (and probably in Sri Lanka) could be due to several reasons. A non-immune cohort of people, born prior to 1984, who have never been properly vaccinated or had the natural infection were probably the base population for this outbreak. Another group of susceptible people are those who received a single dose of MCV between 1984 and 2000 and missed the booster dose in supplementary programmes. Based on previous data, the proper antibody response could not have happened in 15 % of the people who received only a single dose of the MCV [14]. Among those who received two doses, a decrease in vaccine effectiveness may have contributed to this outbreak.

In the initial phase of the outbreak, mainly infants were affected. This could be attributed to several factors. Recent studies have shown that babies born to mothers who were immune from measles due to vaccination rather than natural infection are more susceptible to infection unless they are immunized early. This is due to babies’ relatively low antibody level (or short period of passive immunity) that has transferred from the mother’s immunity due to being vaccinated [15, 16]. Most importantly, however, is the question of whether the shift of the first dose of MCV being administered at 9 months of age to 12 months had a significant effect on the current outbreak. Typically, transferred immunity from mother to baby starts to wane around the age of 5 to 9 months. If mothers have vaccine-derived immunity, waning starts much earlier [17]. Recent studies also point to early susceptibility to measles in infants of both vaccinated women and women with naturally acquired immunity [18]. In Sri Lanka, the mean age of women at childbirth is around 28 years. At the time of this outbreak, some mothers in this age group may not have ever had measles and therefore transferred the antibody to their babies; around 50 % might have had vaccine-derived immunity. Even among vaccinated women, antibody levels could be low [19]. Either way, infants born to these mothers are more susceptible to measles in early infancy. Even though vaccine effectiveness is 95 % when administered at the age of 12 months [20], the chances of getting measles could be reduced by giving the vaccine at the age of 9 months, as this is around the age when risk of mortality is higher. Cost benefits and health outcomes would be much better if the first dose of MCV was administered at 9 months, even though its effectiveness rate is lower (85 %) [21]. The change in the vaccine schedule means that infants are now susceptible to measles. It could have been the triggering factor for this outbreak, as it could have increased the cohort of susceptible population up to the threshold level for the outbreak.

Measles can be associated with complications such as pneumonia, otitis media, encephalitis, diarrhoea and rare long-term sequel subacute sclerosing pan encephalitis. During the 1999–2000 outbreak, only 2.2 % of patients had features suggestive of pneumonia. Meanwhile, diarrhoea, otitis media and encephalitis were noted in 16.7, 1.4 and 0.5 % of patients, respectively. Neurological complications of measles during this outbreak were well documented [22]. There were five measles-related deaths, however, their actual causes were not documented. Among the 33 patients admitted to the UMU THA, 30 (90.9 %) and 26 (78.8 %) had pneumonia and diarrhoea, respectively. Other complications were not observed, but two patients became tachypnoeic and hypoxic and required ICU care. Most of the measles patients admitted to the UMU had a normal WCC, thrombocytopenia and elevated liver transaminases, which can also be noted in dengue fever, Rickettsial infections, atypical pneumonia and sometimes leptospirosis, all of which are common in Sri Lanka. Therefore, if the rash goes unnoticed, especially in dark-skinned individuals, measles can be mistakenly treated as one of the above conditions.

## Conclusion

In developing countries such as Sri Lanka, better health indicators have been developed due to exceptional public health programmes such as the current immunization programme. However, these indicators aren’t necessarily related with an improvement in the socio-economic status, as poverty, poor hygiene, overcrowded environments and other risk factors for communicable disease transmission exist in these countries. This may be the primary reason for communicable disease outbreaks in developing countries, even those triggered by minor factors. According to the analysis of data from the THA, the triggering factor for this outbreak may have been the change in the immunization schedule in 2012. However, it is essential to investigate national data from Sri Lanka to arrive at a final conclusion. This information may be extremely important on a global scale for future decision-making, in order to eradicate measles once and for all.

## Additional file

Additional file 1:
**Multilingual abstracts in the six official working languages of the United Nations.** (PDF 387 kb)

## Abbreviations

ELISA: enzyme-linked immunosorbent assay
EPI: expanded programme on immunization
ICU: intensive care unit
MCV: measles-containing vaccine
MRI: Medical Research Institute
THA: Teaching Hospital, Anuradhapura
UMU: university medical unit
WCW: white cell count
