# Supplementary material for: The 2013 measles outbreak in Sri Lanka: experience from a rural district and implications for measles elimination goals
Source: Infect Dis Poverty. 2015 Nov 30;4:51. doi: 10.1186/s40249-015-0084-7 (PMC4667465; doi:10.1186/s40249-015-0084-7)
Supplement: Additional file 1: — Multilingual abstracts in the six official working languages of the United Nations. (PDF 387 kb) [file 40249_2015_84_MOESM1_ESM.pdf]

Translation of the abstract into the six official working languages of the United Nations

تفشي داء الحصبة في سيريلانكا في العام 2013: الخبرة المكتسبة من إحدى المقاطعات الريفية، والآثار المترتبة على مَهمّة القضاء على داء الحصبة

نيروشانا جاثون داهاناياكا [1-2]، سيثوميني باهالاجاماج [3]، رانجان مادوشانكا جانيجاما [4]، براسانا ويراوانسا [1]، سونيث بودهيكا أجامبودي [2-4]*

ملخص

معلومات تمهيدية: استطاعت سيريلانكا في العام 2011 أن تكون أول دولة في منطقة جنوب شرق آسيا تنجح في القضاء على داء الحصبة. وفي العام 2012، جرى تغيير جدول التلقيح ضد الحصبة، فبدلاً من أن يكون لقاحاً مفرداً ضد الحصبة يُعطى بعمر 9 أشهر، أصبح لقاحاً ثلاثياً ضد الحصبة والنكاف والحصبة الألمانية ويُعطى بعمر 12 شهراً. ولكن في العام 2013 أعلنت سريلانكا عن حدوث تفشٍ للإصابة بالحصبة، اعتبرته الأسوأ من نوعه.

تحرّت هذه الدراسة تفشي الحصبة في إحدى مناطق سيريلانكا بهدف تفسير وشرح أسباب هذه الوبائية.

الطرائق: تم إجراء دراسة استشرافية في الوحدة الطبية الجامعية في مشفى أنورادهابورا التعليمي THA، والذي يُعد ثالث أضخم مشفى في سيريلانكا، وذلك الفترة بين أكتوبر 2013 ومارس 2014. وقد جرى رسم نموذج وبائي للمرضى، والتحري عن جميع الحالات المشتبه بها، وجمع البيانات السكانية (الديموغرافية) لهذه الحالات من السجلات المتوفرة.

النتائج: جرى قبول 101 حالة حصبة مشتبه بها في المشفى التعليمي بأنورادهابورا، وذلك الفترة الممتدة بين يناير 2103 و مارس 2014. بحلول يونيو 2013 كانت جميع الحالات المشتبه بها دون عمر 12 شهراً. أما خلال كامل فترة الدراسة (15 شهراً) فقد توزعت الشرائح العمرية للمرضى على الشكل التالي:

أقل من 9 أشهر: 10 إصابات (9.9%)

9-12 شهر: 11 إصابة (10.9%)

1-11 سنة: 6 إصابات (5.9%)

12-29 سنة: 37 إصابة (36.6%)

أكثر من 29 سنة: 36 إصابة (35.6%)

من بين 33 مريض مشتبه بإصابته من خلال الفحوص السريرية، أثبتت التحاليل المخبرية إصابة 32 شخصاً منهم بالحصبة. وتضمنّت العلامات السريرية للمرضى الثلاثة والثلاثين: الحمى (33 حالة، 100% من الحالات)، الاندفاع الجلدي البُقعي (33 حالة)، التهاب الملتحمة (23 حالة)، اعتلال العقد اللمفية الرقبية الخلفية (23 حالة)، بقع كوبليك (8 حالات). شوهدت علامات تشير إلى الإصابة بالتهاب رئوي لدى 30 حالة (90.9%)، وعانى 26 مريضاً (78.8%) من الإسهال. وقد جرت معالجة اثنين (6.1%) من المرضى الذين أصيبوا بالتهاب رئوي شديد في وحدة العناية المركزة في المستشفى بسبب الصعوبات التنفسية. من بين 33 مريضاً، كان 15 مريضاً (45.5%) قد تلقوا لقاحاً ضد الحصبة، وادعى اثنان من المرضى (6.1%) بأنهما لم يحصلا أبداً على أيّ لقاح ضد الحصبة، في حين كان 16 من المرضى (48.5%) غير متأكدين من حصولهم أو عدم حصولهم على لقاح الحصبة. ومن بين المرضى الذين قالوا بأنهم تلقوا لقاح الحصبة كان 11 منهم (73.3%) ينتمون إلى الفئة العمرية 12-29 سنة.

الاستنتاج: بما أن الحالات الأولية المصابة في هذا التفشي كانت جميعها لأطفال رُضّع، فمن الممكن عزو التفشي الأخير للحصبة جزئياً إلى التغير الحاصل في جدول اللقاحات الذي أدّى بدوره إلى زيادة أعداد الأطفال المعرضين للإصابة بالحصبة.

Translated from English version into Arabic by Sari M. Barazi, through

TRANSLATORS
WITHOUT BORDERS

2013 年斯里兰卡麻疹暴发：农村地区的个案经验及对达到麻疹消除目标的启示

Niroshana Jathun Dahanayaka, Sithumini Pahalagamage, Ranjan Madushanka Ganegama, Prasanna Weerawansa, Suneth Buddhika Agampodi

摘要

引言：2011 年斯里兰卡成为东南亚地区第一个达到麻疹消除目标的国家。2012 年，斯里兰卡麻疹免疫接种程序从对 9 月龄婴儿进行单独麻疹疫苗接种改为对 12 月龄婴儿进行麻疹、腮腺炎和风疹三联疫苗接种。然而，2013 年斯里兰卡报告了近来最严重的一次麻疹暴发。本研究对这次暴发进行了部分调查，以期对其流行病学进行阐述。

方法：本研究以 2013 年 10 月至 2014 年 3 月，在斯里兰卡第三大医院——阿努拉德普勒教学医院（THA）的大学医疗部进行了一项前瞻性调查。构建了患者流行病学数据库。对所有疑似病例进行了核实。从已有记录中获取疑似病例的基本人口学信息。

结果：从 2013 年 1 月到 2014 年 3 月，THA 收治了 101 例麻疹疑似病例。截至 2013 年 6 月，所有疑似病例均为 12 月龄以下婴儿。本次调查期间（15 个月），收治的 9 月龄以下、9-12 月龄、1-11 岁、12-29 岁、29 岁以上患者数分别为 10 例(构成比为 9.9%，下同)、11 例(10.9%)、6 例(5.9%)、37 例(36.6%)和 36 例(35.6%)。33 例临床疑似病例中，32 例麻疹检测阳性。主要临床症状包括：发热 (n=33, 100%)、斑丘疹(n=33)、结膜炎(n=31)、后颈部淋巴结病(n=23)和科氏斑（Koplik’s spots，n=8)。30 例(90.9%)患者有肺炎指征，26 例(78.8%)有腹泻。2 例(6.1%)发展为重症肺炎的患者因呼吸困难在重症监护室进行治疗。33 例临床疑似病例中， 15 例(45.5%)曾接种过麻疹疫苗，2 例(6.1%)声称未曾接种过，16 例(48.5%)接种状态不明。在 15 例曾接种者中，11 例(73.3%)患者的年龄段在 12-29 岁间。

结论：本次暴发的首批病例均为婴儿。因此，本次麻疹暴发可部分归因于疫苗接种计划的变更导致易感婴儿数量的上升。

Translated from English version into Chinese by Qian Men-bao, edited by Yang Pin, through

CHINESE CENTER FOR DISEASE CONTROL AND PREVENTION
NATIONAL INSTITUTE OF PARASITIC DISEASES

L’épidémie de rougeole de 2013 au Sri Lanka : expérience dans un district rural et implications pour les objectifs d’élimination de la rougeole

Niroshana Jathun Dahanayaka[1,2], Sithumini Pahalagamage[3], Ranjan Madushanka Ganegama[4], Prasanna Weerawansa[1], Suneth Buddhika Agampodi [2,4*]

Résumé

Contexte : Sri Lanka a été le premier pays d’Asie du Sud-Est à atteindre son objectif d’élimination de la rougeole en 2011. En 2012, le schéma vaccinal est passé d’un vaccin antirougeoleux à l’âge de 9 mois à un vaccin rougeole-oreillons-rubéole à 12 mois. Le Sri Lanka a néanmoins connu sa pire épidémie récente de rougeole en 2013. La présente étude examine une partie de cette épidémie, dans le but d’en faire une description épidémiologique.

Méthodes : Une étude prospective a été menée à l’unité médicale universitaire de l’Hôpital de formation d’Anuradhapura (THA), le troisième hôpital par ordre de taille au Sri Lanka, d’octobre 2013 à mars 2014. Un profil épidémiologique des patients a été élaboré, tous les cas suspects ont fait l’objet de tests de confirmation et les données démographiques de ces cas suspects ont été relevées dans les dossiers disponibles.

Résultats : Entre janvier 2013 et mars 2014, 101 cas suspects de rougeole ont été admis au THA. Jusqu'à juin 2013, tous ces cas suspects étaient âgés de moins de 12 mois. Pendant la durée de l'étude (15 mois), le nombre total de patients dans les différentes tranches d'âge était de: 10 patients âgés de moins de 9 mois (9,9 %), 11 de 9 à 12 mois (10,9 %), 6 de 1 à 11 ans (5,9 %), 37 de 12 à 29 ans (36,6 %) et 36 de plus de 29 ans (35,6 %). Le dépistage de la rougeole s'est avéré positif chez 32 des 33 patients cliniquement suspects. Les symptômes cliniques fréquents étaient de la fièvre (n=33, 100 %), une éruption maculopapulaire (n=33), une conjonctivite (n=31), une lymphadénopathie cervicale postérieure (n=23) et des taches de Koplik (n=8). Des signes suggérant une pneumonie ont été observés chez 30 patients (90,9 %), et 26 patients (78,8 %) avaient des diarrhées. Deux patients (6,1 %) ont développé une pneumonie sévère avec détresse respiratoire et ont été soignés en unité de soins intensifs. Sur ces 33 patients, 15 (45,5 %) avaient été vaccinés contre la rougeole, deux (6,1 %) ont signalé qu'ils n'avaient jamais été vaccinés et 16 (48,5 %) n'étaient pas certains de leur statut vaccinal. Parmi les patients vaccinés, 11 (73,3 %) appartenaient au groupe d'âge de 12 à 29 ans.

Conclusion : Les premiers cas observés au cours de l'épidémie étaient des nourrissons. De ce fait, l'épidémie pourrait en partie s'expliquer par l'augmentation du nombre de jeunes enfants exposés en raison du changement du plan vaccinal.

Translated from English version into French by Suzanne Assenat, through

TRANSLATORS WITHOUT BORDERS

Вспышка кори в 2013 году на Шри-Ланке: опыт из сельской местности и условия, необходимые для ликвидации кори

Нирошана Джатхун Даханаяка[1,2], Ситхумини Патхалагамаж[3], Ранджан Мадушанка Джанеджама[4], Прасанна Вееραванса[1], Сунет Буддхика Агамподи[2,4*]

Резюме

Справочная информация: Шри-Ланка стала первым государством в Юго-Восточной Азии, которому удалось ликвидировать корь в 2011 году. В 2012 году календарь прививок от кори изменился: вместо прививки от кори в 9 месяцев стали делать прививку от кори, паротита и краснухи в 12 месяцев. Но в 2013 году на Шри-Ланке вспыхнула самая острая за всю историю эпидемия кори. Настоящее исследование посвящено этой эпидемии и имеет целью описать ее эпидемиологию.

Методы: Проспективное исследование проводилось в университетской клинике Teaching Hospital, Anuradhapura (THA), третьей по величине клинике Шри-Ланки с октября 2013 года по март 2014 года. Был сконструирован эпидемиологический профль пациентов, подтверждение выполнялось во всех сомнительных случаях, были получены основные демографические детали на основании доступных записей этих подозрений на болезнь.

Результаты: С января 2013 года по март 2014 года в THA поступил 101 пациент с подозрением на корь 101. До июня 2013 все случаи с подозрением на корь наблюдались у пациентов в возрасте менее 12 месяцев. В течение периода исследования (15 месяцев) возраст всех пациентов менее 9 месяцев, 9 – 12 месяцев, 1 – 11 лет, 12-29 лет и более 29 лет был следующим: 10 (9.9%), 11 (10.9%), 6 (5.9%), 37 (36.6%)

и 36 (35.6%), соответственно. 32 из 33 подозрений на корь оправдались. Наблюдались следующие общие клинические симптомы: лихорадка (n=33, 100%), макулопапулезная сыпь (n=33), коньюктивит (n=31), поражение шейных и паховых лимфатических узлов (n=23) и коревая сыпь (n=8). Признаки пневмонии наблюдались у 30 (90.9%) пациентов, у 26 (78.8%) была диарея. У двух пациентов (6.1%) развилась серьезная пневмония, им потребовалась специальная помощь для ликвидации последствий затрудненного дыхания. 15 (45.5%) из 33 пациентов были вакцинированы от кори, 2 (6.1%) сообщили, что никогда не делали прививки от кори и 16 (48.5%) не знали, были ли они вакцинированы. Из тех, кто был вакцинирован от кори, 11 (73.3%) относились к возрастной группе 12–29 лет.

Заключение: Поскольку первыми жертвами этой эпидемии стали дети, то увеличение подозрений на корь и вспышку заболевания можно объяснить изменением расписании вакцинации младенцев.

Translated from English version into Russian by Jekaterina Merkuljeva, through

TRANSLATORS WITHOUT BORDERS

El brote de sarampión en Sri Lanka durante 2013: la experiencia de un distrito rural y sus implicaciones para los objetivos de eliminación del sarampión

Niroshana Jathun Dahanayaka[1,2], Sithumini Pahalagamage[3], Ranjan Madushanka Ganegama[4], Prasanna Weerawansa[1], Suneth Buddhika Agampodi [2,4*]

Resumen

Antecedentes: Sri Lanka fue el primer país del sudeste asiático en lograr la meta de eliminación del sarampión el año 2011. En el 2012 el programa de inmunización para el sarampión cambió, y en vez de una vacuna contra el sarampión a los nueve meses se pasó a una vacuna contra el sarampión, paperas y rubeola a los 12 meses. Sin embargo, en 2013 Sri Lanka informó de su peor brote reciente de sarampión. El presente estudio investigó una parte de este brote a fin de describir su epidemiología.

Métodos: se realizó un estudio de posibilidades en la unidad médica universitaria del Hospital Docente Anuradhapura (THA), el tercer hospital más grande de Sri Lanka, de octubre 2013 a marzo 2014. Se construyó un perfil epidemiológico de los pacientes, se hizo una confirmación de caso en todos los casos sospechosos, y a partir de los registros disponibles se obtuvieron los detalles demográficos básicos de esos casos sospechosos.

Resultados: entre enero 2013 y marzo 2014 se admitieron 101 casos sospechosos de sarampión en el THA. Hasta junio 2013 todos los casos sospechosos tenían menos de 12 meses de edad. Durante el período de estudio (15 meses) el número total de pacientes con menos de nueve meses, nueve a 12 meses, 1 a 11 años, 12 a 29 años y más de 29 años fueron 10 (9.9%), 11 (10.9%), 6 (5.9%), 37 (36.6%) y 36 (35.6%), respectivamente. De 33 pacientes clínicamente sospechosos hubo 32 que fueron positivos a sarampión. Las características clínicas comunes incluyeron: fiebre (n=33, 100%), erupción maculopapular (n=33), conjuntivitis (n=31), linfadenopatía cervical posterior (n=23) y manchas de Koplik (n=8). Se observaron elementos que sugerían neumonía entre 30 (90.9%) pacientes, y 26 (78.8%) tuvieron diarrea. Dos pacientes (6.1%) que desarrollaron una neumonía grave fueron recibidos en una unidad de cuidados intensivos debido a dificultades respiratorias. De 33 pacientes, 15 (45.5%) tuvieron inmunización previa para sarampión, dos (6.1%) informaron que nunca fueron inmunizados para

sarampión, y 16 (48.5%) no estuvieron seguros sobre su situación en cuanto a inmunización. Entre los que informaron haber sido previamente inmunizados, 11 (73.3%) estaban en el grupo de edad de 12–29 años.

Conclusión: puesto que los primeros casos de este brote fueron niños pequeños, el brote podría explicarse parcialmente por un aumento de niños susceptibles debido al cambio en el programa de vacunación.

Translated from English version into Spanish by gaord, through

TRANSLATORS
WITHOUT BORDERS
